# Mutations Associated with No Durable Clinical Benefit to Immune Checkpoint Blockade in Non-S-Cell Lung Cancer

**DOI:** 10.3390/cancers13061397

**Published:** 2021-03-19

**Authors:** Guangsheng Zhu, Dian Ren, Xi Lei, Ruifeng Shi, Shuai Zhu, Ning Zhou, Lingling Zu, Ramon Andrade De Mello, Jun Chen, Song XU

**Affiliations:** 1 Department of Lung Cancer Surgery, Tianjin Medical University General Hospital, Tianjin 300050, China; yueshengzhu@outlook.com (G.Z.); Ren_Dian@126.com (D.R.); woshileixi@outlook.com (X.L.); spark914@foxmail.com (R.S.); Zhushuaitheforce@163.com (S.Z.); xiaozhouning@outlook.com (N.Z.); zulingling@tmu.edu.cn (L.Z.); 2 Tianjin Key Laboratory of Lung Cancer Metastasis and Tumor Microenvironment, Lung Cancer Institute, Tianjin Medical University General Hospital, Tianjin 300050, China; 3 Escola Paulista de Medicina, Federal University of Sao Paulo, Sao Paulo 04037-004, Brazil; 4 Division of Oncology, Algarve Biomedical Centre, Department of Biomedical Sciences and Medicine, University of Algarve, 8005-139 Faro, Portugal

**Keywords:** non-small cell lung cancer, immunotherapy, KEAP1, FAT1, PD-1/PD-L1 inhibitors, anti-PD1/PD-L1, anti-CTLA-4, no durable clinical benefit, NDB

## Abstract

**Simple Summary:**

With the application of immunotherapy in patients with non-small-cell lung cancer (NSCLC), we found that immunotherapy for some patients cannot achieve long-term effects. Therefore, the purpose of this study is to explore the characteristics of these patients and make a model that can effectively predict the prognosis of immunotherapy patients. The results of this study will make it easier for clinicians to screen out NSCLC patients for immunotherapy.

**Abstract:**

(1) Background: The immune checkpoint blockade (ICB) has shown promising efficacy in non-small-cell lung cancer (NSCLC) patients with significant clinical benefits and durable responses, but the overall response rate to ICBs is only 20%. The lack of responsiveness to ICBs is currently a central problem in cancer immunotherapy. (2) Methods: Four public cohorts comprising 2986 patients with NSCLC were included in the study. We screened 158 patients with NSCLC with no durable clinical benefit (NDB) to ICBs in the Rizvi cohort and identified NDB-related gene mutations in these patients using univariate and multivariate Cox regression analyses. Programmed death-ligand 1 (PD-L1) expression, tumor mutation burden (TMB), neoantigen load, tumor-infiltrating lymphocytes, and immune-related gene expression were analyzed for identifying gene mutations. A comprehensive predictive classifier model was also built to evaluate the efficacy of ICB therapy. (3) Results: Mutations in FAT1 and KEAP1 were found to correlate with NDB in patients with NSCLC to ICBs; however, the analysis suggested that only mutation in FAT1 was valuable in predicting the efficacy of ICB therapy, and that mutation in KEAP1 acted as a prognostic but not a predictive biomarker for NSCLC. Mutations in FAT1 were associated with a higher TMB and lower multiple lymphocyte infiltration, including CD8 (T-Cell Surface Glycoprotein CD8)+ T cells. We established a prognostic model according to PD-L1 expression, TMB, smoking status, treatment regimen, treatment type, and FAT1 mutation, which indicated good accuracy by receiver operating characteristic (ROC) analysis (area under the curve (AUC) for 6-months survival: 0.763; AUC for 12-months survival: 0.871). (4) Conclusions: Mutation in FAT1 may be a predictive biomarker in patients with NSCLC who exhibit NDB to ICBs. We proposed an FAT1 mutation-based model for screening more suitable NSCLC patients to receive ICBs that may contribute to individualized immunotherapy.

## 1. Introduction

Lung cancer is the most commonly diagnosed malignancy and the leading cause of cancer-related deaths worldwide [1]. Non-small-cell lung cancer (NSCLC) accounts for 80–85% of all lung cancers [2], and targeted therapy is suitable only in a small proportion of NSCLC patients with actionable driver gene mutations [3]. With the recent rapid development in immunotherapy, immune checkpoint drugs—especially inhibitors of programmed cell death protein 1 (PD-1) and programmed death-ligand 1 (PD-L1)—have made breakthroughs in the treatment of NSCLC, particularly in cases without targetable driver mutations. A number of clinical trials have shown that immune checkpoint blockades (ICBs) can benefit patients undergoing the first- and second-line treatment for advanced NSCLC, consolidation treatment for locally advanced NSCLC, and neoadjuvant treatment for early NSCLC [4,5].

The ICBs show promising efficacy in NSCLC patients with significant clinical benefits and durable responses, but the overall response rate to anti PD-1/PD-L1 therapy is only 20–30% [4,5]. In particular, a heterogeneity of unique response pattern may be observed in patients receiving ICBs, including pseudoprogression and hyperprogression. Pseudoprogression indicates that the tumor size or number of tumor lesions increases initially, followed by a regression in tumor burden that would benefit the patients receiving ICBs treatment [6]. The lack of responsiveness to ICBs is currently a central problem in cancer immunotherapy. Therefore, it is necessary to explore the characteristics of patients who do not benefit from immunotherapy, to decrease medical costs, and allow individualized treatment.

Biomarkers such as PD-L1 expression [7,8], tumor mutation burden (TMB) [9], neoantigen load [10], tumor infiltrating immune and stromal cells [11], and immune-regulatory mRNA expression [12] may be suitable for clinical selection of patients receiving ICBs, but the utility of each marker is limited. In the current study, we aimed to explore the characteristics and underlying mechanisms of poor responsiveness in NSCLC patients who have no durable clinical benefit (NDB) to treatment with ICBs through investigation of public databases [13,14].

## 2. Materials and Methods

### 2.1. Data Sources

Whole-exome sequencing (WES) data of 1144 NSCLC cases from the Cancer Genome Atlas (TCGA) cohort [14] was obtained through cBioPortal (http://www.cbioportal.org/, accessed on 20 September 2020). The RNA-seq data of 515 LUAD and 501 LUSC were downloaded from TCGA (https://portal.gdc.cancer.gov/, accessed on 21 September 2020). Additionally, we obtained the clinical data of the Zehir cohort [15] (*n* = 1567), Rizvi cohort [13] (*n* = 240), and Naiyer cohort [16] (*n* = 35) from the Memorial Sloan Kettering Cancer Center (MSKCC) using cBioPortal (http://www.cbioportal.org/, accessed on 20 September 2020). Detailed information for each cohort is shown in Table 1. The number of neoantigens in the above-mentioned TCGA cohort was obtained from the Cancer Immunome Atlas (https://tcia.at/home, accessed on 24 September 2020). Durable clinical benefit (DCB) was defined as partial response/stable disease that lasted for >6 months. Patients with no durable clinical benefit (NDB) were included in this study. The role of each cohort is shown in Figure S1.

### 2.2. Cox Proportional Hazards Models

We identified 158 patients with NSCLC from the Rizvi cohort who had no durable clinical benefit (NDB) from immunotherapy. The NGS (next-generation sequencing) results we obtained were processed level 3 data, and we extracted mutation data from MAF (Mutation Annotation Format) files using Perl. The genes with high frequency mutation (mutation ratio > 5%) were selected and analyzed by univariate Cox regression. Factors with *p* < 0.2 were retained and corrected by multivariate Cox regression analysis (Table S1). Finally, factors with *p* < 0.05 in multivariate Cox regression analysis were selected for further study. Univariate Cox regression analysis was performed on the selected factors and some common clinical indicators in the Rizvi cohort, such as age, gender, etc. (Table S2). The factors for establishing a multivariate Cox regression model were determined. A calibration curve of the nomogram was constructed for internal verification. The risk score was calculated according to the regression coefficient. Patients were divided into low-risk and high-risk score groups by median risk score. Another dataset, the Naiyer cohort, was used as an external validation cohort to validate the model (Figure S2).

### 2.3. Propensity Score Matching and Survival Analysis

The “matchit” R package was used to match the Zehir cohort’s propensity score. Matching was performed with the use of a 1:3 matching protocol. The patients were divided into two groups by gene mutation or not, and then the baseline data of the two groups were matched. “Survminer” R package was used to analyze the survival of the matched patients, and a Kaplan–Meier plot was drawn.

### 2.4. Assessment of TMB

The TMB was defined as the number of somatic nonsynonymous variations, insertions, and deletions in the examined coding regions of tumor tissues by whole exome sequencing (WES) in TCGA cohort or next-generation sequencing (NGS) in the Rizvi cohort. In the Rizvi cohort, 56 NSCLC samples underwent targeted NGS (i.e., integrated mutation profiling of actionable cancer-related genes) with a customized panel of 341 genes, 164 samples were analyzed with a panel of 410 genes, and 20 samples were analyzed with a panel of 468 genes.

### 2.5. mRNA Expression Profiling Analysis

The response to ICBs is related to immune tumor microenvironments, including T-cell effector, IFN(Interferon)-γ-associated genes, T-cell receptors, and immune factors [17,18]. The associations between some gene mutations and relevant immune-regulatory mRNA expression were analyzed in 1026 patients in TCGA, for whom both RNAseq and DNAseq data were available. The immune gene list was mainly based on a published article that summarized the genes related to activated T cells, immune cytolytic activity, and IFN-γ release [19]. A list of 47 immune-related genes is provided in Table S3.

### 2.6. Functional Enrichment Analysis

The “clusterProfiler” R package was utilized to perform Gene Set Enrichment Analysis (GSEA) to analyze the differences between the wild-type and FAT1/KEAP1 mutation groups. FDR (False Discovery Rate) < 0.25 was considered statistically significant. XCell [20] cell type enrichment analysis from gene expression data for 64 immune and stroma cell types in TCGA cohort was obtained through TIMER2.0 (http://timer.comp-genomics.org/, accessed on 20 September 2020).

### 2.7. Statistical Analyses

Analyses were performed using R version 3.6.2 (R Foundation for Statistical Computing, Vienna, Austria). Package ggplot2, rms, foreign, survival ROC, and survival were used for statistical and graphics analyses, and Package survival and Survminer were used for survival analyses. An independent sample *t*-test was used for comparison between two groups of samples, the Wilcoxon test was used for comparison between multiple groups of samples, and Spearman correlation analysis was used to detect correlations. A *p*-value < 0.05 was considered statistically significant.

## 3. Results

### 3.1. Clinical Characteristics of Cohorts

A total of four reported cohorts were included in this study. The baseline information of the cohorts is shown in Table 1. In the Naiyer cohort, anti-PD-1 or anti-PD-L1 inhibitors were administered in all the patients. In the Rizvi cohort, 206 and 34 NSCLC patients were treated with anti-PD-1/PD-L1 and anti-PD-1/PD-L1 plus anti-CTLA-4, respectively (Table 1). Additionally, there were 142 and 16 NSCLC patients who showed NDB after treatment with anti-PD-1/PD-L1 and anti-PD1/PD-L1 plus anti-CTLA-4, respectively (Table 1).

### 3.2. Identification of Immune NDB Related Genes

**Figure 1:**
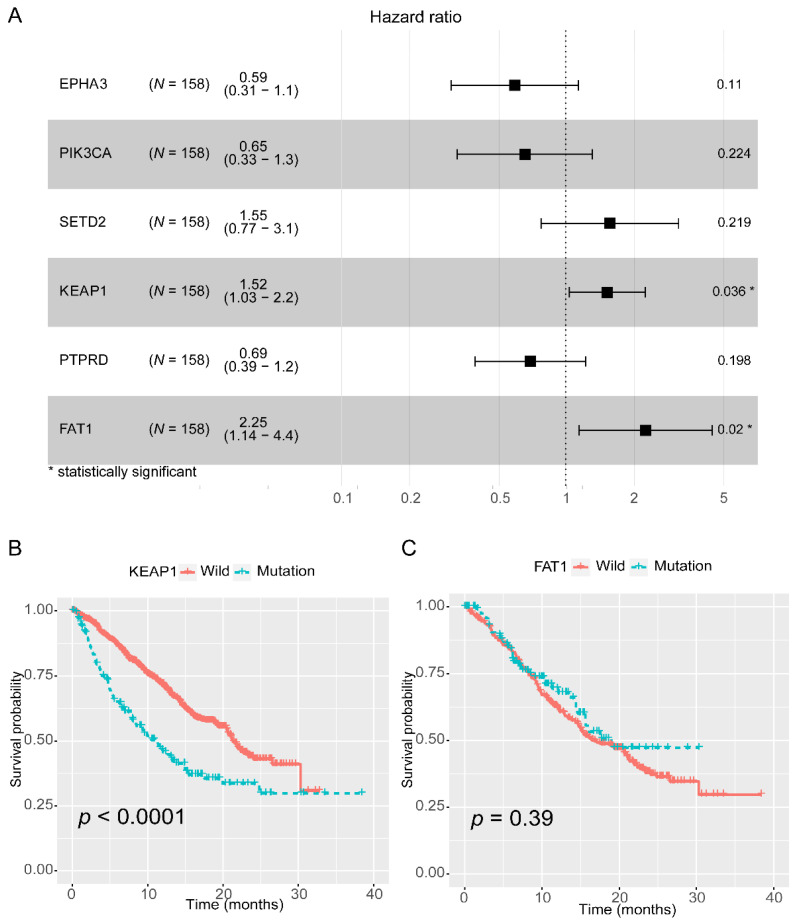
(**A**) Cox proportional hazard analysis to model association of mutations in KEAP1 and FAT1 with prognosis in patients who showed no durable clinical benefit (NDB) to treatment with immune checkpoint blockade (ICB). (**B**) The survival curve for OS (Overall Survival) according to the KEAP1 mutation status in the Zehir cohort. (**C**) The survival curve for OS according to the FAT1 mutation status in the Zehir cohort.

We used the Rizvi cohort to analyze NSCLC patients with NDB (*n* = 158). NDB refers to progressive disease or partial response/stable disease that lasts for <6 months. The genes with high mutation frequency (mutation ratio > 5%) were selected and analyzed by univariate Cox regression. Factors with *p* < 0.2 were retained and corrected by multivariate Cox regression analysis. As shown in Figure 1A, mutations in KEAP1 and FAT1 were significantly associated with a worse prognosis in NSCLC patients with NDB who received ICBs, suggesting that these genes may be negative indicators or are involved in the primary resistance to ICBs. Mutations in KEAP1, but not in FAT1, associate with a worse prognosis in patients with NSCLC without immunotherapy (Figure 1B,C).

### 3.3. Correlation of Immune Phenotypes in NSCLC with Mutations in KEAP1 and FAT1

**Figure 2:**
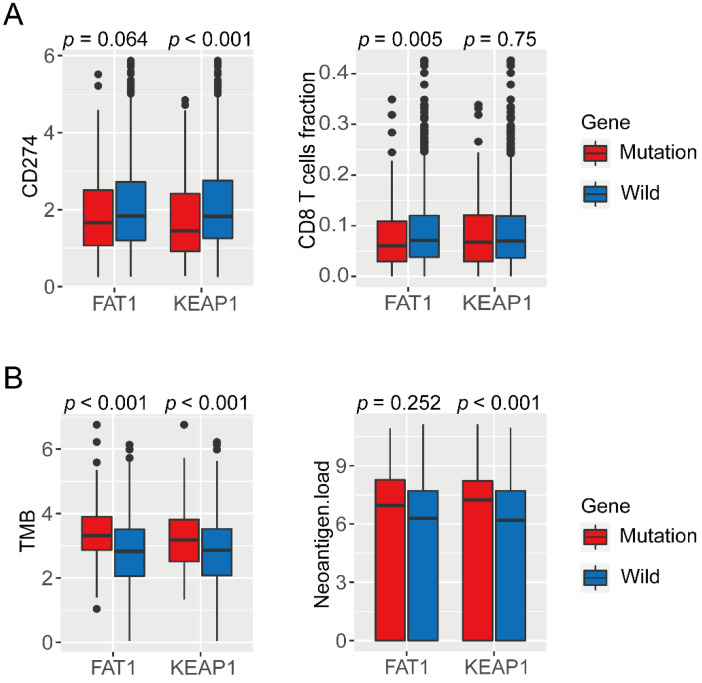
Correlation between gene mutation and status of common biological indicators in patients treated with immune checkpoint blockade (ICB). (**A**) Left: mRNA expression of CD274, right: CD8+ T cell infiltration; (**B**) left: tumor mutation load, right: neoantigen load.

To further explore the underlying mechanism of poor efficacy of ICBs in NSCLC patients with mutations in KEAP1 and FAT1, TCGA cohort was used to compare the differences in immune cell infiltration, neoantigens, TMB, PD-L1 expression, and immune-regulatory mRNA expression between tumors harboring mutations in KEAP1 and FAT1, and those with wild-type genes. The expression of PD-L1 in the KEAP1-mutant NSCLC was significantly lower than that in KEAP1-wild-type NSCLC (*p* < 0.001, Figure 2A). The TMB in NSCLC cases harboring mutations in both the genes was higher than that in cases with wild-type genes (*p* < 0.001, Figure 2B). Moreover, we observed that the tumors with mutation in KEAP1 exhibited more neoantigens than those with wild-type KEAP1 (*p* < 0.001, Figure 2B). In terms of CD8+ T cells, cell infiltration in cases with NSCLC harboring mutation in FAT1 was significantly lower than that in patients with wild-type FAT1 (*p* = 0.005, Figure 2A). Mutations in FAT1 and KEAP1 generated several complex tumor antigens and higher TMB, but as the expression of many immune-related genes was significantly lower, it may lead to immune escape or poor efficacy of ICBs.

**Figure 3:**
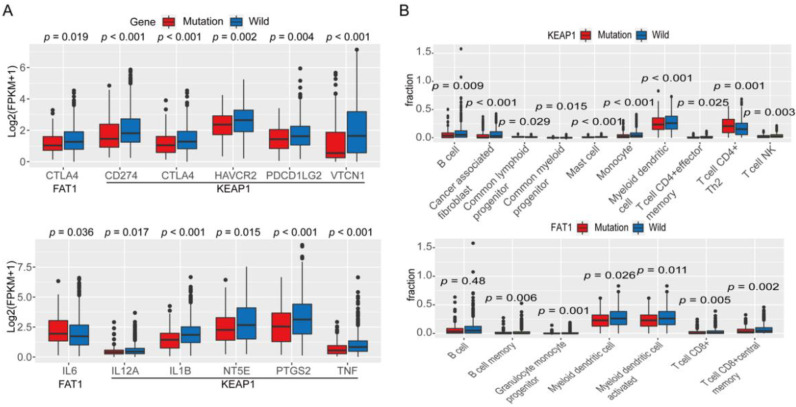
Expression of immune-regulatory genes and status of immune cell infiltration. (**A**) Expression of immune checkpoint genes (upper); status of immune cell infiltration induced by mutation in KEAP1 (lower); (**B**) expression of tumor immune microenvironment genes (upper); status of immune cell infiltration induced by mutation in FAT1 (lower).

In NSCLC cases with mutation in KEAP1, the infiltration levels of eight immune-related lymphocytes were lower, except those of common lymphoid progenitor cells and Th2 (T helper 2) CD4 (T-Cell Surface Glycoprotein CD4)+ T cells, which were higher than levels in cases with wild-type KEAP1 (Figure 3B). In NSCLC cases with mutation in FAT1, the infiltration degree of seven types of immune-related cells was lower than that in cases with wild-type FAT1 (Figure 3B).

**Figure 4:**
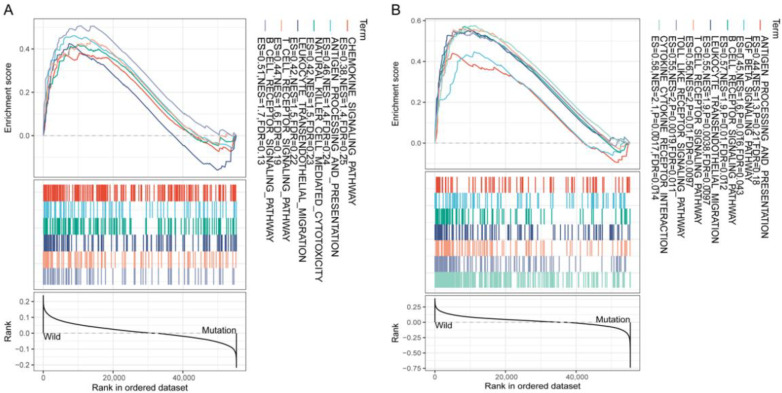
Gene Set Enrichment Analysis (GSEA). (**A**) GSEA between the wild-type and FAT1 mutation groups. (**B**) GSEA between the wild-type and KEAP1 mutation groups.

Moreover, to investigate the correlation between mutations in KEAP1 and FAT1 and the immune-regulatory mRNA expression signature, we analyzed 1026 samples with both RNAseq and DNAseq data in TCGA. Gene set enrichment analysis was conducted between the wild-type and FAT1/KEAP1 mutation groups. The analysis suggested that cases with mutation in FAT1 had concomitant downregulation of several immune-related pathways, including chemokine signaling pathway, antigen processing and presentation, natural killer cell-mediated cytotoxicity, leukocyte transendothelial migration, T-cell receptor signaling pathway, and B-cell receptor signaling pathway (Figure 4A). In cases with mutation in KEAP1, several immune-related pathways were also deregulated, including antigen processing and presentation, TGF-beta(Transforming Growth Factor-β) signaling pathway, leukocyte transendothelial migration, T-cell receptor signaling pathway, B-cell receptor signaling pathway, toll-like signaling pathway, and cytokine–cytokine receptor interaction (Figure 4B).

**Figure 5:**
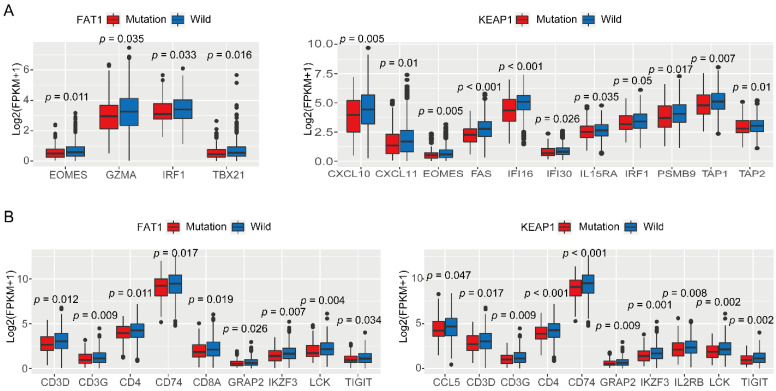
Expression of immune-regulatory genes. (**A**) T-cell effector and IFN-γ-associated gene signatures; (**B**) T-cell receptor (TCR)-related genes.

Furthermore, the NSCLC cases with mutation in FAT1 demonstrated lower levels of the following gene clusters than those in cases with wild-type FAT1: immune checkpoint (CTLA-4, Figure 3A); tumor immune microenvironment (IL6, Figure 3A); T-cell effector and IFN-γ-associated signatures (EOMES, GZMA, GZMB, IRF1, and TBX21; Figure 5A); and T-cell receptor (TCR)-related genes (CD3G, CD3D, CD4, CD8A, CD74, GRAP2, IKZF3, LCK, and TIGIT; Figure 5B). In contrast, in cases with mutation in KEAP1, some gene clusters demonstrated lower levels of mRNA expression than that in cases with wild-type KEAP1, including immune checkpoint (CD274, CTLA-4, HAVCR2, PDCD1LG2, and VTCN1; Figure 3A); tumor immune microenvironment (IL1B, IL12A, NT5E, PTGS2, TNF; Figure 3B); T-cell effector and IFN-γ-associated signatures (CXCL9, CXCL10, CXCL11, GZMA, GZMB, IFNG, IRF1, PMSB9, and TAP1; Figure 5A); and T-cell receptor (TCR)-related genes (CCL5, CD3G, CD3D, CD4, CD74, GRAP2, IKZF3, IL2RB, LCK, and TIGIT; Figure 5B).

### 3.4. Integration of FAT1 Mutation and Clinical Indicators to Predict Prognosis after Treatment with ICBs

**Figure 6:**
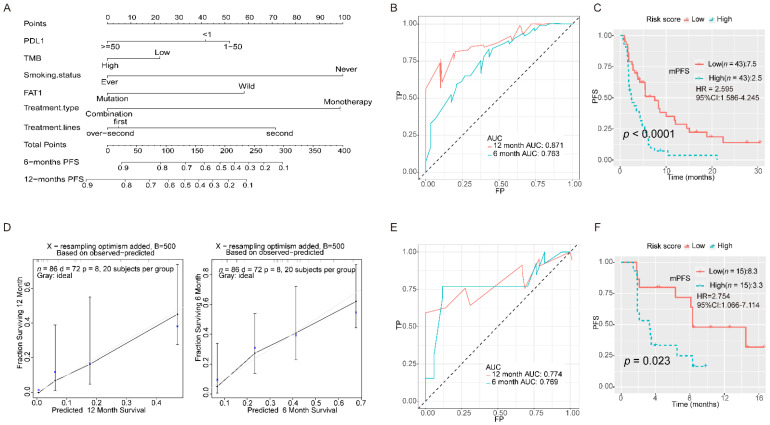
Construction of an integrated prognostic classifier model. (**A**) A nomogram based on PD-L1 expression, tumor mutation burden (TMB), smoking status, treatment regimen, treatment type, and FAT1 mutation status in the Rizvi cohort. (**B**) Receiver operating characteristic (ROC) curves for predicting progression-free survival (PFS) using the nomogram for the Rizvi cohort. (**C**) The PFS curve with the nomogram for the Rizvi cohort. The risk score is calculated according to the regression coefficient. According to the risk score, the cohort is divided into low-risk and high-risk score groups for Kaplan Meier survival analysis. (**D**) Calibration plot of the nomogram to determine the probability of PFS at 6 months (right) and 12 months (left) in the Rizvi cohort. (**E**) ROC curves for predicting PFS of the nomogram in the Naiyer cohort. (**F**) The survival curve for PFS with the nomogram according to the risk score in the Naiyer cohort.

We selected six variables, including PD-L1, TMB, smoking status, treatment regimen, treatment type, and FAT1 mutation status by univariate Cox regression, that were used to establish a prognostic model (Figure 6A). The receiver operating characteristic (ROC) analysis indicated a good accuracy of this model (area under the curve (AUC) for 6-month survival: 0.763; AUC for 12-month survival: 0.871; Figure 6B). Furthermore, the calibration curve for the nomogram was constructed for internal verification (Figure 6D). The survival analysis showed that the patients with low-risk factors had a better progression-free survival (PFS) than those with high-risk factors (2.5 months vs. 7.5 months, *p* < 0.001, hazards ratio (HR): 2.595, 95% confidence interval (CI): 1.586–4.245; Figure 6C). Moreover, we used the Naiyer cohort as an external validation cohort to test the prognosis model. The ROC curve suggested that this immune signature was highly consistent with the ideal model (AUC for 6-month survival: 0.769; AUC for 12-month survival: 0.774; Figure 6E). The survival analysis showed that the low-risk group had a better PFS (3.3 months vs. 8.3 months, *p* = 0.023, HR: 2.754, 95% CI: 1.066–7.114; Figure 6F).

## 4. Discussion

In this study, we identified mutations in NSCLC patients with NDB from treatment with immune checkpoint blockade using several cohorts in public databases. The analyses demonstrated that mutation in FAT1 could be used as a predictive indicator for efficacy of ICBs, while mutation in KEAP1 acted as a prognostic but not predictive biomarker in NSCLC patients receiving treatment with ICB. According to a previous study, TMB, PD-L1 expression, neoantigen, CD8+ T-cell infiltration, and immune-regulatory mRNA expression may influence the efficacy of immunotherapy [21]. Therefore, to analyze the mechanism of worst response to ICB in NSCLC patients with NDB, we systematically explored multiple indicators related to immunophenotypes in patients with NSCLC harboring mutations in KEAP1 and FAT1, including TMB, PD-L1 expression, neoantigen, immune cell infiltration, and immune-regulatory mRNA expression. We found that NSCLC cases with mutation in KEAP1 had lower PD-L1 expression than those with wild-type KEAP1. Cases with mutations in KEAP1 and FAT1 tended to have higher TMB and neoantigen loads but a lower expression of immune-related genes and tumor-infiltrating CD8+ cells than cases with wild-type genes. The lack of CD8+ T-cell infiltration and low expression of immune-related pathway genes could have led to the poor therapeutic effect of immunotherapy. Therefore, patients with NSCLC should be cautiously treated with ICB.

In the study of H. Rizvi et al. [13], they reported that STK11 mutations were significantly clustered in NDB patients compared to the DCB patients. They also found that the elevation of TMB would result in a higher probability of DCB, suggesting that STK11 mutations, and a low TMB, could be a potential cause of NDB. However, in our study, we analyzed the mutations of genes with high mutation frequency (mutation rate >5%) in NDB patients and found that STK11 mutation has no significant relationship with the prognosis of NSCLC patients with NDB who received ICBs. We only found that KEAP1 and FAT1 were significantly associated with a worse prognosis in NSCLC patients with NDB who received ICBs, suggesting that these genes may be negative indicators or are involved in the primary resistance to ICBs. However, further analysis indicated that mutations in KEAP1, but not in FAT1, associate with a worse prognosis in patients with NSCLC without immunotherapy in our study. These data confirmed the report from 2020 *ESMO Open* that KEAP1, as well as the STK11, were the prognostic but not predictive biomarkers of immunotherapy or chemotherapy in NSCLC. In the study of Naiyer Rizvi et al. [16], they also reported that TMB would result in a higher probability of DCB, suggesting that a low TMB could be a potential cause of NDB. This is similar to the study of H. Rizvi et al. In our study, our purpose is to explore the potential causes of worse prognosis in patients with NDB. Therefore, the subjects of our study are the NDB patients. This is different from the studies of H. Rizvi et al. and Naiyer Rizvi et al. Skoulidis et al. [22] reported comparable findings that patients with NSCLC harboring mutation in KEAP1 had a shorter PFS and overall survival (OS) than those without mutation in KEAP1. Further analysis revealed that there was no enhanced clinical benefit in patients with mutations in KEAP1 receiving immunotherapy and chemotherapy than those receiving chemotherapy alone [23]. This indicated that mutation in KEAP1 was a prognostic biomarker in NSCLC regardless of the type of treatment administered, although it could not predict the efficacy of immunotherapy. However, a recent study suggested that FAT1-mutated NSCLC exhibited a more durable clinical benefit and better objective response rate to anti-PD-L1 therapy [24], which is inconsistent with analysis in the current study. This discrepancy may be ascribed to the different patient populations in the current and previous studies. We focused on NSCLC with NDB to ICBs, while previous studies recruited all patients with NSCLC receiving ICBs. Moreover, a recent study proposed that deletion of FAT1 may cause upregulated EMT (Epithelial Mesenchymal Transition) status, tumor stemness, and metastatic ability in lung squamous cell carcinoma [25]. This may also explain the poor efficacy of NSCLC to ICB treatments.

Using the Cox regression analysis, we confirmed that FAT1 had a significant predictive effect for ICB treatment in patients with NSCLC. Therefore, we integrated PD-L1 expression, TMB, smoking status, treatment regimen, treatment type, and FAT1 mutation status to establish a clinical prognostic model for ICB treatment, which was further validated using another independent cohort. Furthermore, a nomogram was constructed based on this multivariate Cox regression model. This predictive model showed that the AUC for 12-month ROC curve was 0.871, indicating very good accuracy of the model for predicting long-term PFS. The median of the risk scores calculated from the risk coefficients of this model divided the population into low-risk and high-risk groups, and patients in the low-risk group had a significantly longer PFS than those in high-risk group. This nomogram could help clinicians predict the efficacy of ICBs for the treatment of NSCLC.

However, this study has some limitations. First, as a retrospective study based on public databases, some information such as performance status, treatments prior to ICB, and rescue treatment after disease progression are unavailable, which may cause study bias. Second, the sample size of the included cohorts was relatively small. Finally, not all gene mutations were involved in the analysis because the gene panel was analyzed by targeted NGS rather than WES, and we only focused on high-frequency gene mutations (more than 5%) according to the previous study.

## 5. Conclusions

In conclusion, mutation in FAT1 may be a potential biomarker in NSCLC patients who exhibit NDB to ICB. We propose an FAT1 mutation-based model for screening NSCLC patients who may benefit from treatment with ICB, which may contribute to individualized immunotherapy.

## Figures and Tables

**Table 1:** Clinical characteristics of the included cohorts.

Characteristic		TCGA Cohort *n* = 1144	Rizvi Cohort *n* = 240			Naiyer Cohort *n* = 35	Zehir Cohort *n* = 1567
			DCB	NDB	NA		
Gender	Male	673 (59%)	32 (13%)	79 (33%)	7 (3%)	16 (46%)	681 (43%)
	Female	468 (41%)	37 (15%)	79 (33%)	6 (3%)	19 (54%)	886 (57%)
Age	>60	695 (71%)	39 (16%)	53 (22%)	12 (5%)	19 (54%)	NA
	≤60	253 (26%)	30 (13%)	105 (44%)	1 (0.4%)	16 (46%)	NA
Smoking status	Ever	976 (85%)	60 (26%)	123 (80%)	10 (4%)	30 (86%)	972 (62%)
	Never	111 (10%)	9 (4%)	35 (20%)	3 (1%)	5 (14%)	334 (21%)
	Unknown	57 (5%)	0	0	0	0	261 (17%)
Histology	AD	660 (58%)	56 (23%)	124 (6%)	7 (3%)	30 (86%)	1268 (81%)
	SCC	484 (42%)	9 (4%)	22 (9%)	3 (1%)	4 (11%)	163 (10%)
	Others	0	5 (2%)	12 (5%)	3 (1%)	1 (3%)	136 (9%)
ICBs	PD1/PDL1	NA	53 (22%)	142 (59%)	11 (5%)	35 (100%)	NA
	Combination	NA	16 (7%)	16 (7%)	2 (0.8%)	0	NA

## Data Availability

The Zehir cohort, Rizvi cohort, and Naiyer cohort can be obtained from cBioPortal (http://www.cbioportal.org/, accessed on 20 September 2020). TCGA cohort can be downloaded from the Cancer Genome Atlas (https://portal.gdc.cancer.gov/repository, accessed on 21 September 2020). The data of neoantigens in TCGA cohort can be downloaded from the Cancer Immunome Atlas (https://tcia.at/home, accessed on 24 September 2020).

## Supplementary Materials

The following are available online at https://www.mdpi.com/2072-6694/13/6/1397/s1, Figure S1: Flow chart of the study, Figure S2: Flow chart of clinical prognosis model for immune checkpoint blockade (ICB), Table S1: List of immune-related genes, Table S2: Univariate Cox regression analysis to identify mutation in genes that may be related to worse prognosis in patients with NDB, Table S3: Univariate Cox regression analysis to identify the prognostic factors of ICBs.
