# Supplementary material for: Mutations Associated with No Durable Clinical Benefit to Immune Checkpoint Blockade in Non-S-Cell Lung Cancer
Source: Cancers (Basel). 2021 Mar 19;13(6):1397. doi: 10.3390/cancers13061397 (PMC8003499; doi:10.3390/cancers13061397)
Supplement: Supplementary file 1 [file cancers-13-01397-s001.zip › cancers-1118855-supp/supplement/Figure s1.pdf]

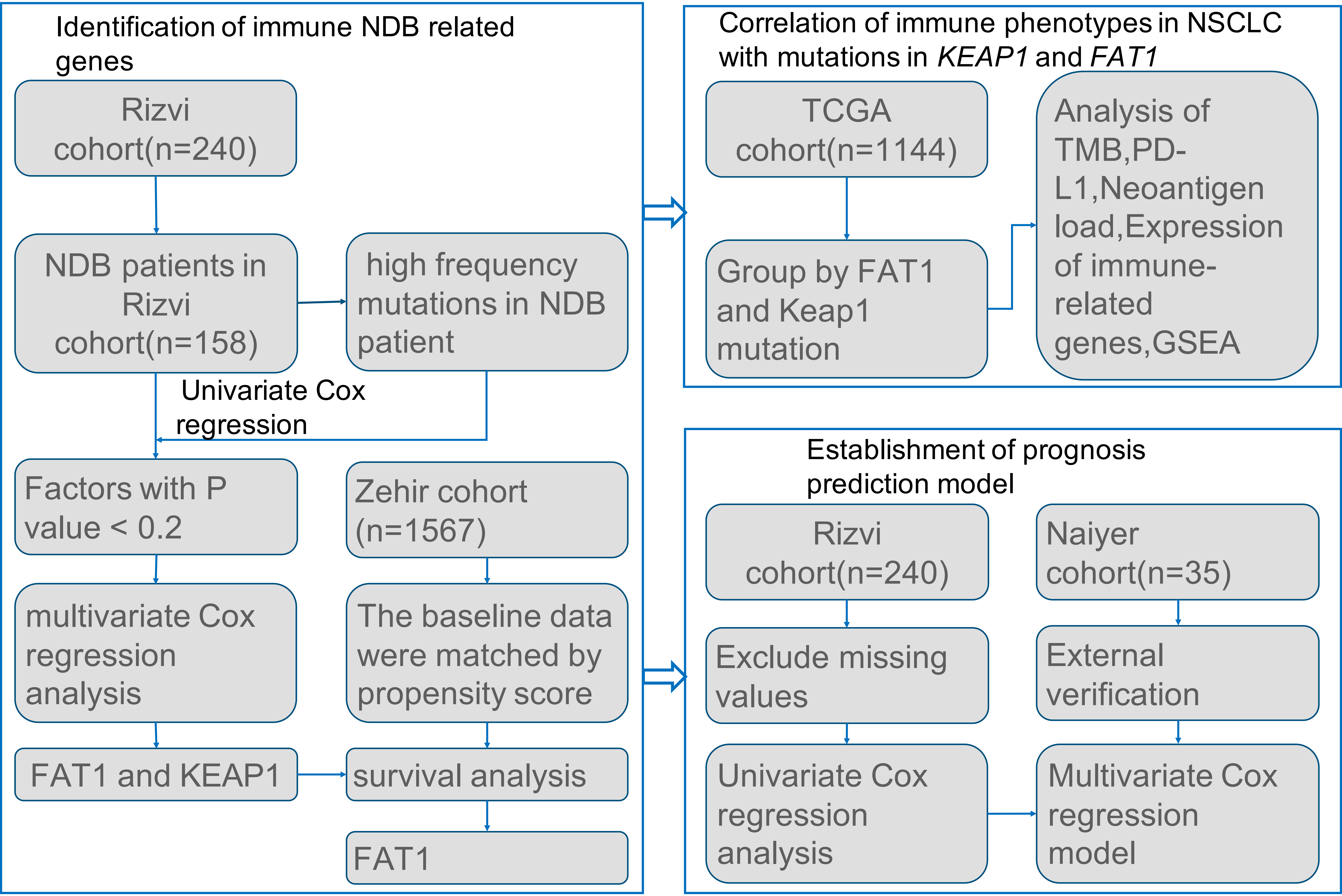
Identification of immune NDB related genes
Rizvi cohort(n=240)
NDB patients in Rizvi cohort(n=158)
high frequency mutations in NDB patient
Univariate Cox regression
Factors with P value < 0.2
Zehir cohort (n=1567)
multivariate Cox regression analysis
The baseline data were matched by propensity score
FAT1 and KEAP1
survival analysis
FAT1
Correlation of immune phenotypes in NSCLC with mutations in KEAP1 and FAT1
TCGA cohort(n=1144)
Group by FAT1 and Keap1 mutation
Analysis of TMB,PD-L1,Neoantigen load,Expression of immune-related genes,GSEA
Establishment of prognosis prediction model
Rizvi cohort(n=240)
Naiyer cohort(n=35)
Exclude missing values
External verification
Univariate Cox regression analysis
Multivariate Cox regression model
