# Supplementary material for: Mutations Associated with No Durable Clinical Benefit to Immune Checkpoint Blockade in Non-S-Cell Lung Cancer
Source: Cancers (Basel). 2021 Mar 19;13(6):1397. doi: 10.3390/cancers13061397 (PMC8003499; doi:10.3390/cancers13061397)
Supplement: Supplementary file 1 [file cancers-13-01397-s001.zip › cancers-1118855-supp/supplement/Table S1.docx]

**Table S1.** Univariate Cox regression to identify mutation genes that might be related to worse prognosis in patients with NDB

| id | HR | HR.95L | HR.95H | pvalue |
| --- | --- | --- | --- | --- |
| STK11 | 0.938314 | 0.652301 | 1.349735 | 0.731423 |
| KEAP1 | 1.310264 | 0.893545 | 1.921326 | 0.16647 |
| EGFR | 1.076004 | 0.672074 | 1.722704 | 0.760318 |
| RBM10 | 0.987161 | 0.59531 | 1.636939 | 0.960059 |
| PTPRD | 0.695891 | 0.413561 | 1.170961 | 0.172084 |
| SMARCA4 | 1.250248 | 0.733104 | 2.132193 | 0.412197 |
| MLL2 | 0.904364 | 0.521386 | 1.568653 | 0.720537 |
| NF1 | 0.773955 | 0.446354 | 1.342 | 0.361523 |
| ARID1A | 1.350545 | 0.760481 | 2.398447 | 0.305105 |
| EPHA5 | 0.857568 | 0.485434 | 1.514979 | 0.59665 |
| MLL3 | 1.050885 | 0.594662 | 1.857119 | 0.864347 |
| ATM | 1.376577 | 0.757912 | 2.500243 | 0.293889 |
| EPHA3 | 0.566558 | 0.311844 | 1.029322 | 0.062168 |
| PTPRT | 0.749707 | 0.414665 | 1.355457 | 0.340389 |
| FAT1 | 1.535998 | 0.82845 | 2.847836 | 0.173041 |
| NOTCH4 | 1.191545 | 0.64387 | 2.20507 | 0.576809 |
| PIK3CG | 1.233764 | 0.66702 | 2.282051 | 0.503194 |
| CDKN2A | 0.92169 | 0.479977 | 1.7699 | 0.806488 |
| CDKN2Ap16INK4A | 0.92169 | 0.479977 | 1.7699 | 0.806488 |
| HGF | 0.761539 | 0.400677 | 1.447402 | 0.405739 |
| PIK3CA | 0.561452 | 0.293814 | 1.072886 | 0.080631 |
| SETD2 | 1.592982 | 0.833967 | 3.042797 | 0.15851 |
| SMAD4 | 1.25439 | 0.658849 | 2.388247 | 0.490265 |
| CREBBP | 1.231638 | 0.624965 | 2.427228 | 0.547224 |
| PBRM1 | 1.310121 | 0.664968 | 2.581203 | 0.434976 |
| RB1 | 1.059273 | 0.538906 | 2.082108 | 0.867368 |
| ALK | 0.970739 | 0.475842 | 1.980353 | 0.934934 |
| APC | 1.125498 | 0.551006 | 2.298969 | 0.745615 |
