# Supplementary material for: Mutations Associated with No Durable Clinical Benefit to Immune Checkpoint Blockade in Non-S-Cell Lung Cancer
Source: Cancers (Basel). 2021 Mar 19;13(6):1397. doi: 10.3390/cancers13061397 (PMC8003499; doi:10.3390/cancers13061397)
Supplement: Supplementary file 1 [file cancers-13-01397-s001.zip › cancers-1118855-supp/supplement/Table S2.docx]

**Table S2.** Univariate Cox regression analysis to identify the prognostic factors of ICBs

| id | HR | HR.95L | HR.95H | pvalue |
| --- | --- | --- | --- | --- |
| AGE | 0.954949 | 0.751363 | 1.213696 | 0.706306 |
| Sex | 0.814514 | 0.51164 | 1.296677 | 0.387138 |
| PDL1 | 0.988989 | 0.981356 | 0.996682 | 0.005099 |
| TMB | 0.587266 | 0.36309 | 0.94985 | 0.03003 |
| Smoking.status | 0.476618 | 0.273855 | 0.829508 | 0.008764 |
| Treatment.lines | 1.424237 | 1.07591 | 1.885335 | 0.013464 |
| Treatment.Type | 0.374231 | 0.173856 | 0.805544 | 0.011978 |
| KEAP1 | 0.771815 | 0.427333 | 1.393988 | 0.390501 |
| FAT1 | 0.467854 | 0.222875 | 0.98211 | 0.044678 |
