# Supplementary material for: Mutations Associated with No Durable Clinical Benefit to Immune Checkpoint Blockade in Non-S-Cell Lung Cancer
Source: Cancers (Basel). 2021 Mar 19;13(6):1397. doi: 10.3390/cancers13061397 (PMC8003499; doi:10.3390/cancers13061397)
Supplement: Supplementary file 1 [file cancers-13-01397-s001.zip › cancers-1118855-supp/supplement/Table S3.docx]

**Table S3.** List of immune-related genes

| category | GENE |
| --- | --- |
| Immune checkpoint | PDCD1 |
| Immune checkpoint | CD274 |
| Immune checkpoint | PDCD1LG2 |
| Immune checkpoint | LAG3 |
| Immune checkpoint | CTLA4 |
| Immune checkpoint | HAVCR2 |
| Immune checkpoint | VTCN1 |
| T-effector and INFγ pathway | GBP1 |
| T-effector and INFγ pathway | IFI16 |
| T-effector and INFγ pathway | IFI30 |
| T-effector and INFγ pathway | IFNG |
| T-effector and INFγ pathway | IRF1 |
| T-effector and INFγ pathway | STAT1 |
| T-effector and INFγ pathway | TAP1 |
| T-effector and INFγ pathway | TAP2 |
| T-effector and INFγ pathway | FAS |
| T-effector and INFγ pathway | PSMB9 |
| T-effector and INFγ pathway | IL15RA |
| T-effector and INFγ pathway | GZMA |
| T-effector and INFγ pathway | GZMB |
| T-effector and INFγ pathway | EOMES |
| T-effector and INFγ pathway | CXCL10 |
| T-effector and INFγ pathway | CXCL9 |
| T-effector and INFγ pathway | CXCL11 |
| T-effector and INFγ pathway | TBX21 |
| T-effector and INFγ pathway | PRF1 |
| T cell receptor | CD27 |
| T cell receptor | GRAP2 |
| T cell receptor | LCK |
| T cell receptor | PTPRCAP |
| T cell receptor | CCL5 |
| T cell receptor | IL2RB |
| T cell receptor | IKZF3 |
| T cell receptor | CD3G |
| T cell receptor | CD74 |
| T cell receptor | CD3D |
| T cell receptor | CD8A |
| T cell receptor | CD4 |
| T cell receptor | TIGIT |
| Tumor microenvironment | IDO1 |
| Tumor microenvironment | PTGS2 |
| Tumor microenvironment | IL1B |
| Tumor microenvironment | IL18 |
| Tumor microenvironment | IL6 |
| Tumor microenvironment | IL12A |
| Tumor microenvironment | TNF |
| Tumor microenvironment | NT5E |
