# Supplementary figures and images for: Mutations Associated with No Durable Clinical Benefit to Immune Checkpoint Blockade in Non-S-Cell Lung Cancer
Source: Cancers (Basel). 2021 Mar 19;13(6):1397. doi: 10.3390/cancers13061397 (PMC8003499; doi:10.3390/cancers13061397)

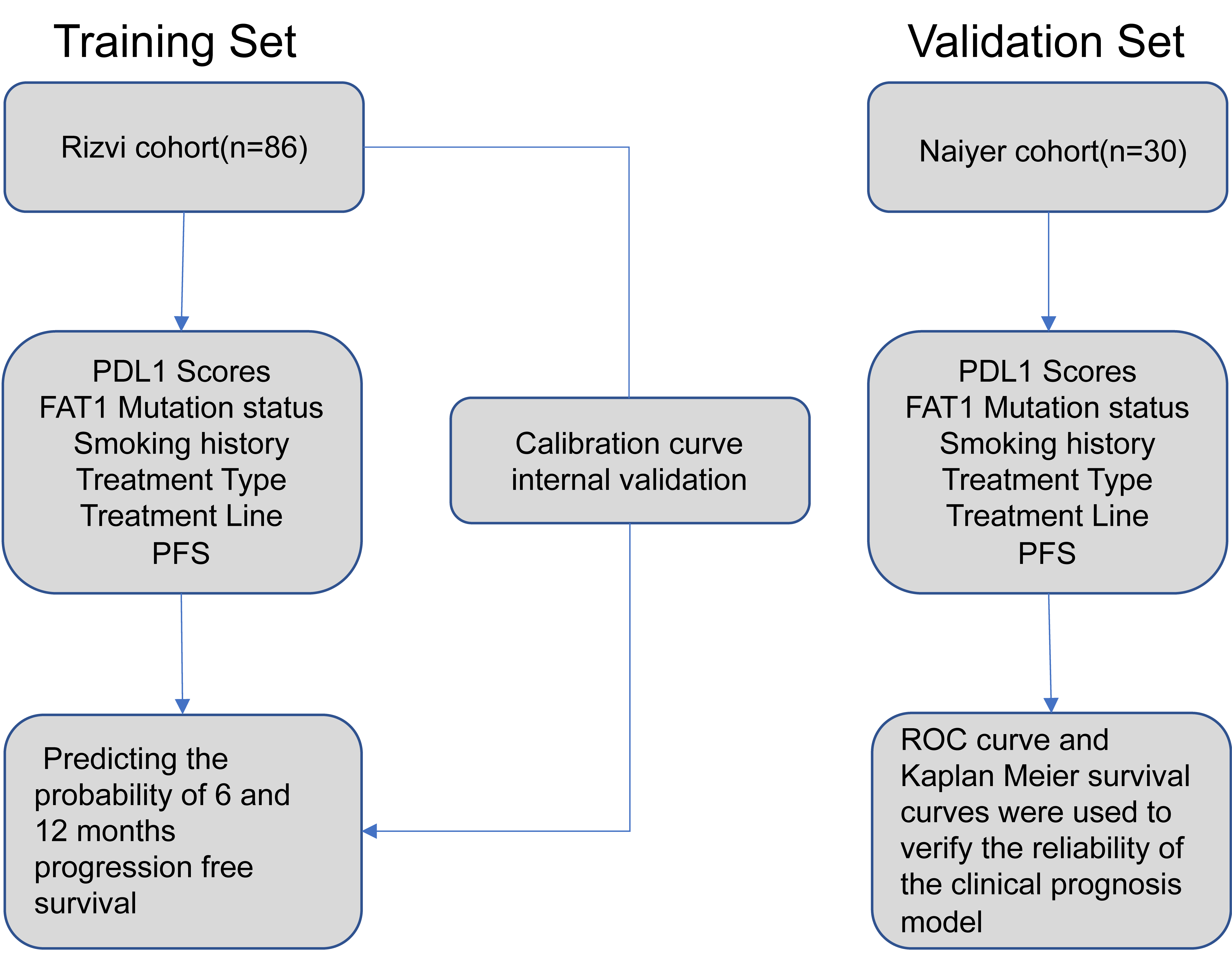

Supplement: Supplementary file 1 [file cancers-13-01397-s001.zip › cancers-1118855-supp/supplement/Figure S2.tif]
